# The Effect of the Isomeric Chlorine Substitutions on the Honeycomb-Patterned Films of Poly(x-chlorostyrene)s/Polystyrene Blends and Copolymers via Static Breath Figure Technique

**DOI:** 10.3390/ma12010167

**Published:** 2019-01-07

**Authors:** Leire Ruiz-Rubio, Leyre Pérez-Álvarez, Julia Sanchez-Bodón, Valeria Arrighi, José Luis Vilas-Vilela

**Affiliations:** 1 Grupo de Química Macromolecular (LABQUIMAC) Dpto. Química-Física, Facultad de Ciencia y Tecnología, Universidad del País Vasco (UPV/EHU), 48940 Leioa, Bizkaia, Spain; leyre.perez@ehu.eus (L.P.-Á.); jsanchez903@ikasle.ehu.eus (J.S.-B.); joseluis.vilas@ehu.eus (J.L.V.-V.); 2 BCMaterials, Basque Center for Materials, Applications and Nanostructures, UPV/EHU Science Park, 48940 Leioa, Spain; 3 Chemical Sciences, School of Engineering & Physical Sciences, Heriot-Watt University, Edinburgh EH14 4AS, UK; V.Arrighi@hw.ac.uk

**Keywords:** poly(x-chlorostyrene), honeycomb, breath figures, conformational entropy

## Abstract

Polymeric thin films patterned with honeycomb structures were prepared from poly(x-chlorostyrene) and statistical poly(x-chlorostyrene-co-styrene) copolymers by static breath figure method. Each polymeric sample was synthesized by free radical polymerization and its solution in tetrahydrofuran cast on glass wafers under 90% relative humidity (RH). The effect of the chorine substitution in the topography and conformational entropy was evaluated. The entropy of each sample was calculated by using Voronoi tessellation. The obtained results revealed that these materials could be a suitable toolbox to develop a honeycomb patterns with a wide range of pore sizes for a potential use in contact guidance induced culture.

## 1. Introduction

The control over water condensing phenomenon is a useful approach to form highly ordered honeycomb structures from polymer by condensation of water droplets onto a drying polymer solution. These honeycomb polymer thin films have attracted much attention due to the increasing range of applications, such as energy storage [1,2], membranes [3,4,5,6,7], catalytic surfaces [8,9], and sensing materials [10], among others. The effectiveness and low cost of these methods in comparison to other techniques, such as photolithography or templating methods, have stimulated interest on breath figures to fabricate substrates for biological applications [11,12,13].

**Figure 1:**
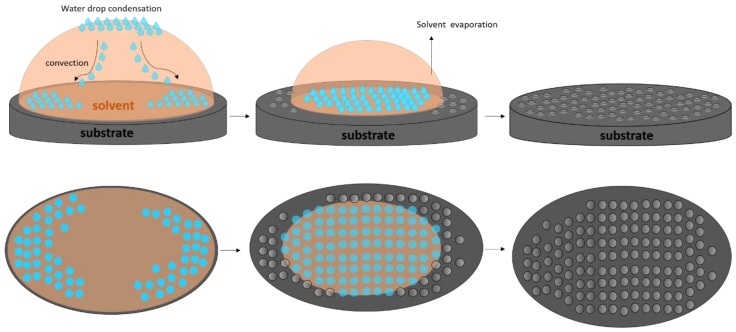
Mechanism of breath figure formation.

In brief, the breath figures were formed when a polymer solution in a high volatile solvent was cast onto a substrate under adequate humidity (Figure 1). The evaporation of the solvent induced a cooling at the solvent/air interface. This process favored the condensation of water from the humidity in the solution. Condensed water droplets formed a honeycomb pattern on the surfaces [14].

**Figure 2:**
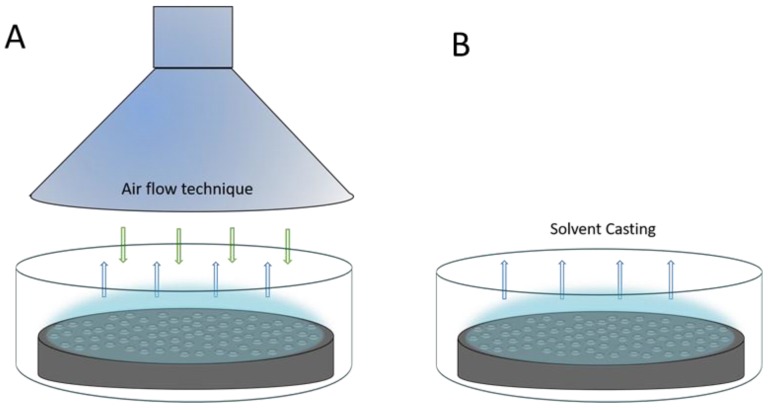
(**A**) Air flow or dynamic technique, and (**B**) solvent cast or static technique.

The formation of the breath figures could be performed by different approaches, such as dip coating, spin coating, air-flow or dynamic technique, and solvent cast or static method, with the last two techniques more extended (Figure 2). On the one hand, in the static breath figure technique, a polymer solution is cast dropwise on a solid substrate under a controlled relative humidity, where the experiment is placed in a close chamber that allows the control over temperature and RH. On the other hand, in the dynamic method, the polymer casting is carried out under airflow, with controlled flow and humidity. This flow forces the rapid evaporation of the solvent due to the formed temperature gradient between the solution and the bulk [15,16]. In this study, a static breath figure method was used.

Several factors could affect the breath figure method, such as humidity, solvent, concentration, polymer, or substrate, among others. For example, an increase on the RH usually induces an increase on the pore size, whereas a solvent with a higher volatility produces a lower pore size. These effects were summarized in several reviews devoted to breath figures [17,18].

Cell-material interaction is considered one of the fundamental fields in biomaterials [19,20,21]. Apart from the chemical composition of the surfaces, physical factors, such as pore size and stiffness, are important in cell adhesion, spreading, and proliferation [22]. Studies indicate that a reduction of the cell adhesive sites on a substrate could be a key factor in the design of the surface structure of cell culture substrates [11]. In this context, recently 2D and 3D patterns have been fabricated, from submicrometer to less than one hundred of micrometer, capable of modeling in vivo topographic microenvironments of cells inducing the contact guidance [20,23,24,25].

Several researchers have demonstrated the successful adhesion of the most common cells to honeycomb structures [26,27,28,29,30]. It has been demonstrated as a promotion of the adhesion for most common cells when using honeycomb patterns, including hepatocytes [28,31] or endothelial cells [27,32], among others. As an example, Arai et al. [33] have reported the influence of the pore size on the incubation of cardiac myocytes. They fabricated poly(ε-caprolactone)–based honeycomb structures with diameters ranging from 4 to 13 µm. This range could be divided in subcellular (around 4.00 µm), cellular (8 µm), and overcellular (12.5 µm), by comparison with the cellular size (7–10 µm for cardiac myocytes). These authors observed that the pore size could crucially affect both cellular adhesion and morphologies. Similar results were reported by Tsuruma and co-workers for neural cell stems [12,34]. Considering these honeycomb patterns could be used to successfully grow several types of cells. However, some studies also reported an excellent growth when unidirectional/anisotropic patterns were used as scaffolds [35]. Besides, the cost of this technique, added to it ease to perform, could arise interest in its industrial fabrication.

The present study reports the fabrication and characterization of highly ordered honeycomb films of polystyrene and poly(x-chlorostyrene)s, their copolymers, and polystyrene/poly(x-chlorostyrene) blends by using the static breath figure method. The polymer solutions were cast on glass wafers under 90% relative humidity (RH). We show that the use of different chlorine substitutions allowed varying the pore size of the surface, in order to obtain topographies with subcellular, cellular, and overcellular diameter (compared to an average cellular diameter of 7 µm), as a potential toolbox in the development of surfaces for contact guidance induced culture. The polystyrene was chosen as a reference material since it is a very commonly used polymer and not expensive, so its use as a template could be highly interesting from an industrial point of view. On the other hand, the synthetic process of these chloro-substituted polymers is quite similar to the polystyrene, and their synthesis could be considered cost-effective when compared to the polymers that have been used for breath figure applications obtained from time–consuming reactions, such as atom transfer radical polymerization or reversible addition–fragmentation chain-transfer polymerization.

## 2. Materials and Methods

### 2.1. Materials

Styrene (S), 2-chlorostyrene (2ClS), 3-chlorostyrene (3ClS), 4-chlorostyrene (4ClS), and the initiator, α,α′-azobisisobutyronitrile (AIBN), were obtained from Sigma Aldrich. Polystyrene (PS, weight-average molecular weight (M_w_) = 3 × 10^6^ g·mol^−1^) was purchased from Polysciences (Warrington, PA, USA). Tetrahydrofuran, HPLC grade, (Scharlab, Sentmenat, Spain) was used without further purification. The polymer solutions were cast in round glass coverslips of 20 mm diameter purchased from Marienfeld (Lauda-Königshofen, Germany).

### 2.2. Synthesis of the Homopolymers and Random Copolymers

Phenolic inhibitors from the 2, 3 or 4-chlorostyrene monomers were first removed by washing with sodium hydroxide (0.1 M) solution, then with hydrochloric acid solution, and finally, with distilled water until neutral pH. The monomers were dried over anhydrous MgSO_4_ and filtered. The initiator, AIBN, was purified by crystallization from methanol.

Homopolymers and statistical copolymers of styrene and x-chlorostyrenes were prepared by free radical bulk polymerization, at 60 °C, under nitrogen atmosphere, with 0.5 mol% AIBN with respect to the total amount of monomer. Copolymers were synthesized at low conversions to prevent composition drifts.

The polymers were precipitated into excess methanol, re-dissolved in chloroform, and re-precipitated in methanol. Finally, the purified samples were vacuum dried at 70 °C for two days.

The copolymer compositions were determined by Fourier Transform Infrared spectroscopy (FTIR) and Elemental Analysis. FTIR spectra were recorded using a Nicolet Nexus FTIR spectrophotometer (Thermo Fisher Scientific, Loughborough, UK), in KBr pellets with a resolution of 4 cm^−1^, and a total of 32 scans were averaged in all cases. The copolymer compositions were obtained from the FTIR data collected for the copolymers, using a calibration curve obtained by measuring the absorbance ratio of two characteristic peaks for each homopolymer, for known homopolymer composition mixtures. Elemental Analysis was carried out using the Eurovector EA3000 (Eurovector, Milan, Italy) analyzer. Copolymer compositions were obtained from the chlorine content.

Molecular weights of all polymer samples were determined by gel permeation chromatography (GPC), using a Waters chromatograph (Mildford, MA, USA) and THF as the eluent. M_w_ values reported in Table 1 and Table 2 are relative to polystyrene standards.

### 2.3. Preparation of the Films

The polymer solutions were prepared by dissolution of the homopolymers or copolymers in THF. The polymer concentration used in this study was 30 mg mL^−1^. The films were prepared from these solutions by casting (50 µL) onto glass wafers under controlled humidity in a closed chamber. The relative humidity (RH) was controlled by a saturated salt solution of KNO_3_ in water to obtain 90% RH. Round films of 150 ± 2 mm diameter and 0.04–0.05 mm thickness were obtained.

### 2.4. Characterization

The morphology of the honeycomb patterned films on a glass substrate was studied by a scanning electron microscopy (SEM). The samples were coated with gold, prior to the SEM measurements, using a Fine Coat Ion Sputter JFC-1100 (JEOL, Tokyo, Japan). SEM micrographs were taken using a Hitachi S-4800 (Hitachi, Tokyo, Japan). The images were processed and analyzed using image analysis freeware ImageJ to obtain pore size (mean diameter) and size distribution of the patterned surfaces. The regularity of the obtained honeycomb-like patterns shown in the SEM images was evaluated by Voronoi tessellation of the images, also performed by ImageJ.

## 3. Results

In this study, the influence of the chlorine substitution and the geometrical positioning of the Cl substituent on the topography and conformational entropy of the honeycomb surfaces were studied. Highly ordered patterns were successfully fabricated from polystyrene and its derivatives by the static breath figure method, using THF as the solvent at 90% RH, at room temperature. The regularity of the patterned films was quantified by measuring the conformational entropy calculated by Voronoi polygons. This method has not only been used to assess the regularity of the surface patterns, but also to describe defects in the membranes [36,37,38].

A Voronoi polygon is defined as the smallest convex polygon surrounding a point whose sides are perpendicular bisectors of the lines between a point and its neighbors [39]. The conformational entropy, which can be related to the degree of order of the arrays, is defined as (Equation (1)):

(1) $$S=-\sum_{n}P_{n}\;\ln P_{n}$$

where *n* is the coordination number of each Voronoi, i.e., the number of sides of the polygon, and *P_n_* is the fraction of the polygons having the coordination number *n*. That is, Voronoi tessellation method calculates the probability of the occurrence of four (*P*_4_), five ((*P*_5_), six—which is a perfectly hexagonal lattice—(*P*_6_), seven (*P*_7_), or eight (*P*_8_) As a reference, for a perfectly ordered hexagonal array, i.e., an ideal hexagonal lattice, the conformational entropy is 0, whereas the entropy of a completely random pattern is 1.71 [18,39,40,41,42,43].

The formation of the patterns was carried out by exploiting the static breath figure method from different samples at 30 mg mL^−1^ and 90% RH. First, the formation of honeycomb arrays in poly(x-chlorostyrene) and polystyrene homopolymers were studied. After, the formation in the patterns obtained for random copolymers of poly(x-chlorostyrene-co-styrene) and, finally, their blends of the polystyrene and poly(x-chlorostyrene) were analyzed.

### 3.1. Formation of Breath Figures in Poly(x-chlorostyrene) Isomers and Polystyrene

The poly(x-chlorostyrene)s were synthesized by free radical polymerization (0.5 mol% AIBN, 60 °C). The weight average molecular weights (M_w_) and the polydispersity index (M_w_/M_n_) of poly(x-chlorostyrene) and polystyrene used in this study were determined by GPC and are summarized in Table 1.

**Figure 3:**
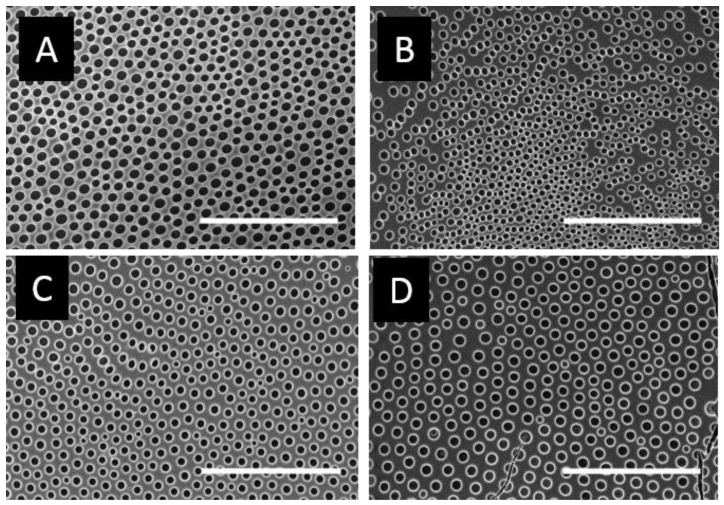
SEM images for (**A**) poly(styrene), (**B**) poly(2-chlorostyrene) (**C**) poly(3-chlorostyrene), and (**D**) poly(4-chlorostyrene). (Scale bar = 100 µm).

The homopolymer samples were cast on a glass substrate, and the obtained arrays were analyzed by SEM. As shown in Figure 3, all samples present cavities at this condition. However, honeycomb patterns based on poly(x-chlorostyrene) samples seem to be less homogenous than polystyrene. These changes could be induced by the change on the chlorine substitution, so different parameters, such as pore size or surface entropy, have been analyzed to evaluate these variations.

The pore diameter and their standard deviation (SD) for x-chloro-substituted samples were 4.1 ± 0.45, 5.25 ± 0.35 and 6.26 ± 0.20 µm for poly(2-chlorostyrene) (P2ClS), poly(3-chlorostyrene) (P3ClS) and poly(4-chlorostyrene) (P4ClS), respectively. Thus, pore size increases from ortho to para substitution, with the cavity diameter of the 4-chloro-substitued polymer similar to that of polystyrene, 6.15 ± 0.38 µm. Size distribution diagrams are presented in Figure S1 of the Supporting Information (SI).

Several authors reported that an increase of the polymer molecular weight usually leads to larger pore sizes [44,45]. However, in our poly(x-chlorostyrene) systems there was no significant variation between the diameters of the polystyrene and the chlorine substituted polymers. That is, the presence of the chloro-substitution could have increase the pore size, due to the influence of the side groups, avoiding the use of high molecular weight polystyrene samples to obtain similar pore size.

**Figure 4:**
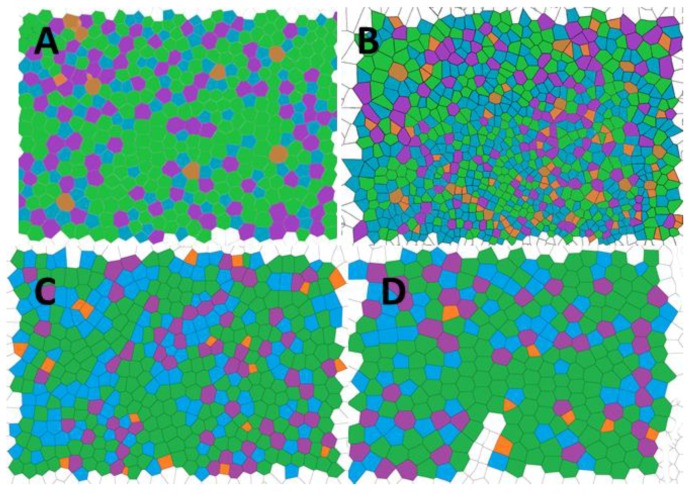
Voronoi tessellation for (**A**) poly(styrene), (**B**) poly(2-chlorostyrene) (**C**) poly(3-chlorostyrene), and (**D**) poly(4-chlorostyrene). (Orange = P_4_, Blue = P_5_, Green = P_6_, Purple = P_7_, Brown = P_8_).

The conformational entropy based on the Voronoi tessellation (Figure 4) indicated that the maximum order was reached for poly(4-chlorostyrene), with a value of entropy equal to 1.035, similar to that obtained for polystyrene (S = 1.033). The other two isomers showed higher entropy values, being equal to 1.294 and 1.112 for P2ClS and P3ClS, respectively. As expected, the irregular chemical structure of the polymer leads to highly disordered structures. The chlorine substituent in poly(4-chlorostyrene) samples are in the less hindered position, and this makes P4ClS similar to polystyrene, considering both pore size and conformational entropy, even if the molecular weight of the 4-chloro-substituted homopolymer is one magnitude order lower than molecular weight of polystyrene.

### 3.2. Formation of Breath Figures with Poly(x-chlorostyrene-co-styrene) Copolymers

Similar to the homopolymers, statistical copolymers of x-chlorostyrene and styrene, i.e., poly(x-chlorostyrene-co-styrene) were synthesized by free radical polymerization (0.5 mol% AIBN, 60 °C), at the average molecular weight of poly(x-chlorostyrene)s and the fraction of styrene (F_s_) in the copolymer, summarized in the Table 2.

**Figure 5:**
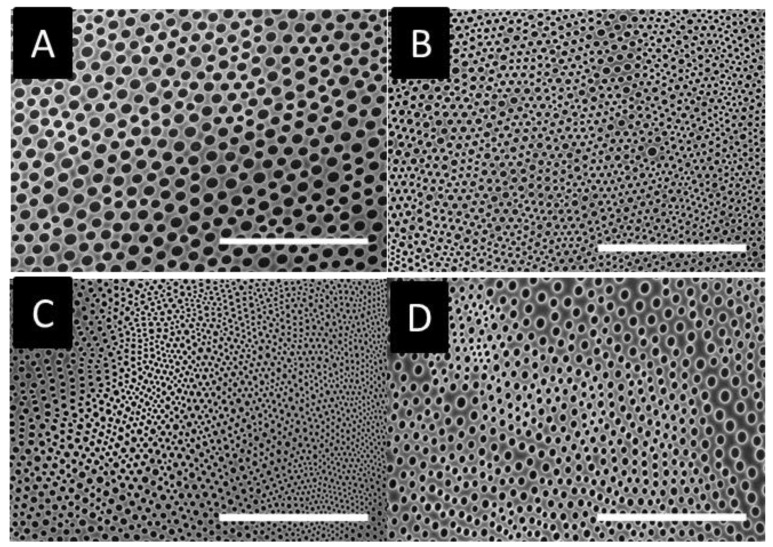
SEM images for (**A**) poly(styrene), (**B**) poly(2-chlorostyrene-co-styrene), (**C**) poly(3-chlorostyrene-co-styrene), and (**D**) poly(4-chlorostyrene-co-styrene). (Scale bar = 100 µm).

The SEM images of the breath figure arrays for the statistical copolymers are compared to polystyrene in Figure 5. As shown in this Figure, the patterns obtained from the copolymers were more homogeneous compared to those of the corresponding homopolymers. As for the homopolymers, polystyrene and poly(4-chlorostyrene-co-styrene) (P(4Cl-co-S)) present more similarities between them.

All of the patterns obtained from the copolymers showed a significant decrease in pore diameter when compared to the homopolymers (Table 3). It is important to notice the improvement in the homogeneity of the honeycomb arrays of the copolymers based on 2-chlorostyrene and 3-chlorostyrene (Figure 5B,D) when compared with those formed from the homopolymers (Figure 3). In addition, these two copolymers with greater steric hindrance groups (with ortho and metha substitutions) presented similar molecular weight, slightly below of the 4-chloro-substituted copolymer, which in this case could have enhanced the slight reduction on pore size. The size distribution diagrams (Figure S2 in the Supplementary) showed a broad distribution that also affected the standard deviation of the pore size diameter that could be attributed to the aforementioned decrease in the size. Homopolymer and copolymer samples presented a good pore distribution in the film surface lower than several cell sizes (commonly, around 10 µm), and these kinds of patterns have been successfully used by several authors for a contact guidance in a cellular growth [12,23,33,34].

**Figure 6:**
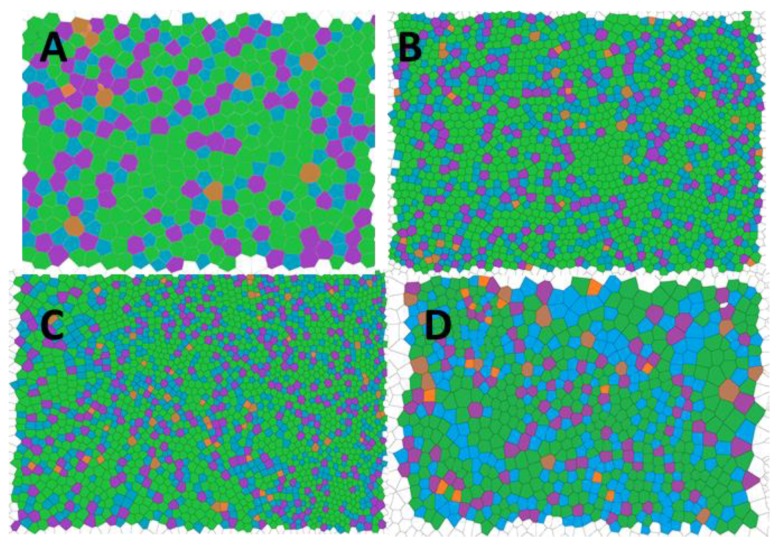
Voronoi tessellation for (**A**) poly(styrene), (**B**) poly(2-chlorostyrene-co-styrene), (**C**) poly(3-chlorostyrene-co-styrene), and (**D**) poly(4-chlorostyrene-co-styrene). (Orange = P_4_, Blue = P_5_, Green = P_6_, Purple = P_7_, Brown = P_8_).

The reduction of the pore size causes a drastic increase in the number of holes present in the patterns. This fact could induce an increase in the disorder of the system. In order to evaluate the influence of the chloro-substitution on the disorder of obtained structures, the conformational entropy was calculated from the Voronoi tessellation (Figure 6). Values are tabulated in Table 4 and compared to those of the homopolymers.

The reduction in pore size positively affected the conformational entropy when P(2ClS-co-S) and P(3ClS-co-S) was studied. For these copolymers, the order of the obtained patterns increased significantly when compared to the homopolymer samples. However, the variation on the 4-chlorostyrene–based samples presented an opposite behavior, and a considerable increase on the entropy was obtained, so in these samples the reduction in the pore size induced an increase on the surface disorder.

The octanol–water partition coefficient (*P*_ow_) is a well-known indicator of the hydrophobicity of an organic compound that could be used for comparing the benzene and chlorobenzene substitution. These compounds present a *P*_ow_ of 2.16 and 2.84, respectively [46,47]. The higher hydrophobicity of the x-chlorosubstituted samples could increase the interface tension, inducing a reduction in the drop size and, therefore, a reduction in the pore size when compared to the polystyrene. In addition, this effect is more relevant to compounds where substitution results in greater steric hindrance.

### 3.3. Formation of Breath Figures in Poly(x-chlorostyrene) Isomers and Polystyrene Blends

**Figure 7:**
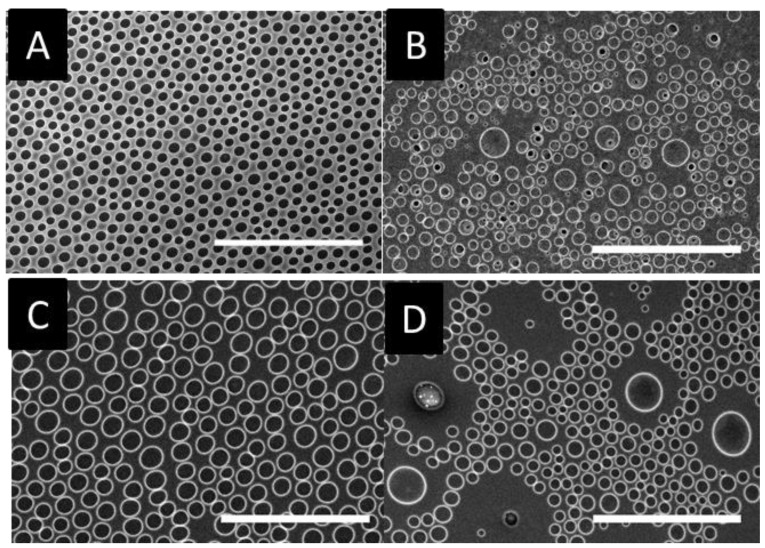
SEM images for (**A**) poly(styrene) and blends (90/10) (**B**) polystyrene/poly(2-chlorostyrene, (**C**) polystyrene/poly(3-chlorostyrene), and (**D**) polystyrene/poly(4-chlorostyrene). (Scale bar = 100 µm).

Several authors have reported the use of blends to develop breath figures [48,49,50]. In view of this, blends of polystyrene with poly(x-chlorostyrene) homopolymers were used to fabricate breath figure patterns. A 90/10 PS/poly(x-chlorostyrene) composition was chosen, considering that most commonly used approach for breath figure used a polymer matrix (polystyrene) and an additive polymer in order to vary the formed patterns [9,49,51,52]; in this case, poly(x-chlorostyrenes)s were used as additives. Both polymers are miscible [53,54] and their miscibility was analyzed by Differential Scanning Calorimetry (DSC) and the obtained glass transition temperatures were summarized in the Supporting Information (Tables S1–S3). However, as shown in Figure 7, even if the polymers were miscible at this composition, the obtained patterns were less regular when compared to the polystyrene matrix. Therefore, this approach, traditionally used for several systems to improve the honeycomb patterns, was not adequate for these materials.

However, a regular pattern was obtained for polystyrene/poly(3-chlorostyrene) blends, with a pore size of 10.62 ± 1.53 μm and conformational entropy equal to 1.18 (Figure S3). The entropy value of this sample was similar to that reported for the homopolymers, but the pore size was found to be even higher than for polystyrene. This discrepancy on the behavior of P3ClS has been also observed when properties of P2ClS, P3ClS and P4ClS were studied. In this line, investigations devoted to the study of local dynamic of poly(x-chlorostyrene)s reported by Casalini et al. [55] described that segmental relaxations depend on the chloro-substitution in the phenyl ring, leading to unexpected results for 3-chlorostyrene. Indeed, this work asserts that the reorientation of pendant of P4ClS was independent of the movement in the main chain of the polymer, and for P2ClS, the rotation of the side group was restricted by the coupling to rotation of the backbone. However, when P3ClS was evaluated, clear results could not be observed, given the constraints of the pendant group intermediate, between P2ClS and P4ClS. In our case, it was observed (Figure 7) that this side group induced a change in the water/polymer interaction. In the case of PS/P2ClS, the lack of mobility of the 2-chloro substitution prevented a uniform rearrangement of the chains forming irregular patterns (Figure 7B). On the other hand, the absence of hindrance for PS/P4ClS blends induced the coalescence of the water droplets forming domains with higher pore size and other domains with smaller pores (Figure 7D). However, in the case of PS/P3ClS, similar to the behavior observed for dielectric relaxation, an intermediate characteristic of 3-chloro substitution allowed the formation of regular arrays (Figure 7C). Moreover, the patterns obtained for polystyrene/poly(3-chlorostyrene) (Figure 7C) presented an average pore size, higher than 10 μm, that could be useful for the culture of larger cells [27].

In order to apply this procedure to larger areas, it is important to notice that the use of raw materials, such as homopolymers or statistical copolymers, leads to homogeneous surfaces of adequate diameter range to be used for future cell cultures. However, in these systems, contrary to many other studies, the use of a polymer blend was not successful. Thus, x-chlorosubstitution and random copolymerization are preferred routes rather than traditional approaches, such as blending.

## 4. Conclusions

In the present study, a series of honeycomb films presenting different pore sizes and conformational entropies were developed using the static breath figure method. This simple film fabrication technique added to the variety in pores’ sizes, making these systems highly suitable for cell culture uses.

## Figures and Tables

**Table 1:** Molecular weight of the studied polymer samples.

Sample	M_w_ (10^4^) (g mol^−1^)	M_w_/M_n_
Polystyrene	300	1.8
Poly(2-chlrostyrene)	23.3	1.3
Poly(3-chlorostyrene)	17.1	1.5
Poly(4-chlorostyrene)	11.7	1.2

**Table 2:** Molecular weight and fraction of styrene in the copolymer for the different samples.

Sample	M_w_ (10^4^) (g mol^−1^)	M_w_/M_n_	F_S_
Polystyrene	300	1.80	1
Poly(2-chlrostyrene-co-styrene)	11.3	1.9	0.526
Poly(3-chlorostyrene-co-styrene)	11.2	1.6	0.608
Poly(4-chlorostyrene-co-styrene)	13.9	1.5	0.619

**Table 3:** Pore diameter of poly(x-chlorostyrene-co-styrene) samples compared to the homopolymers.

Copolymer Sample	Pore Diameter (µm)		Homopolymer Sample
Polystyrene	6.15 ± 0.38		Polystyrene
P(2ClS-co-S)	3.47 ± 0.44	4.1 ± 0.45	P2ClS
P(3ClS-co-S)	2.9 ± 0.46	5.25 ± 0.35	P3ClS
P(4ClS-co-S)	4.36 ± 0.55	6.26 ± 0.20	P4ClS

**Table 4:** Pore diameter of poly(x-chlorostyrene-co-styrene) samples compared with the homopolymers.

Copolymer Sample	Conformational Entropy		Homopolymer Sample
Polystyrene	1.033		Polystyrene
P(2ClS-co-S)	1.061	1.291	P2ClS
P(3ClS-co-S)	1.089	1.112	P3ClS
P(4ClS-co-S)	1.133	1.035	P4ClS

## Supplementary Materials

The following are available online at http://www.mdpi.com/1996-1944/12/1/167/s1, Figure S1: Pore size distribution in THF at 90% RH for: (A) polystyrene, (B) poly(2-chlorostryrene), (C) poly(3-chlorostyrene) and (D) poly(4-chlorostyrene), Figure S2: Pore size distribution in THF at 90% RH for: (A) polystyrene, (B) poly(2-chlorostryrene-co-styrene), (C) poly(3-chlorostyrene-co-styrene) and (D) poly(4-chlorostyrene-co-styrene), Figure S3: Voronoi tessellation for polystyrene/poly(3-chlorostyrene blend. (Orange = P4, Blue = P5, Green = P6, Purple = P7, Brown = P8). Table S1: Glass transition temperatures of PS and P(xClS)s, Table S2: Glass transition temperatures of P(S-co-xClS)s, Table S3: Glass transition temperatures of PS/P(xClS) blends 90/10.
