# Supplementary material for: The Effect of the Isomeric Chlorine Substitutions on the Honeycomb-Patterned Films of Poly(x-chlorostyrene)s/Polystyrene Blends and Copolymers via Static Breath Figure Technique
Source: Materials (Basel). 2019 Jan 7;12(1):167. doi: 10.3390/ma12010167 (PMC6337389; doi:10.3390/ma12010167)
Supplement: Supplementary file 1 [file materials-12-00167-s001.pdf]

# The Effect of the Isomeric Chlorine Substitutions on the Honeycomb-Patterned Films of Poly(x-chlorostyrene)s/Polystyrene Blends and Copolymers via Static Breath Figure technique

**Leire Ruiz-Rubio [1,2,*], Leyre Pérez-Álvarez [1,2], Jula Sanchez-Bodón [1], Valeria Arrighi [3] and José Luis Vilas-Vilela [1,2]**

[1] Grupo de Química Macromolecular (LABQUIMAC) Dpto. Química-Física, Facultad de Ciencia y Tecnología, Universidad del País Vasco (UPV/EHU), 48940 Leioa, Bizkaia, Spain; leyre.perez@ehu.eus (L.P.-Á.); jsanchez903@ikasle.ehu.eus (J.S.-B.); joseluis.vilas@ehu.eus (J.L.V.-V.)

[2] BCMaterials, Basque Center for Materials, Applications and Nanostructures, UPV/EHU Science Park, 48940 Leioa, Spain

[3] Chemical Sciences, School of Engineering & Physical Sciences, Heriot-Watt University, Edinburgh EH14 4AS, UK; V.Arrighi@hw.ac.uk

* Correspondence: leire.ruiz@ehu.eus; Tel.: +34-946017972

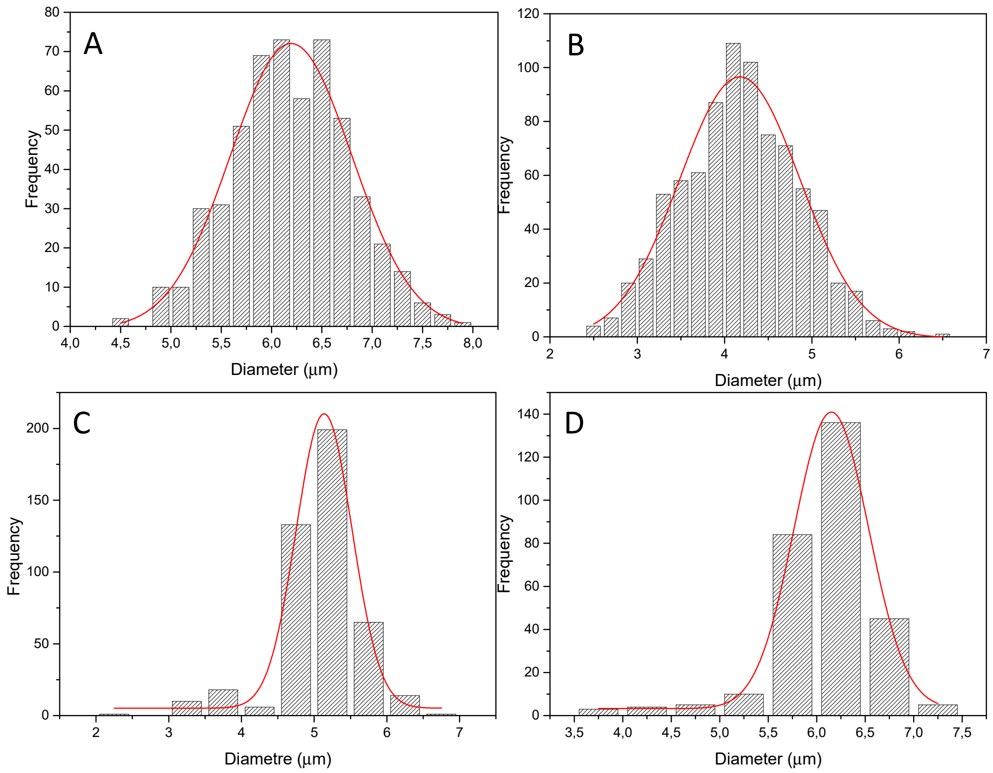


Figure S1. Pore size distribution in THF at 90%RH for: A) polystyrene, B) poly(2-chlorostryrene), C) poly(3-chlorostyrene) and D) poly(4-chlorostyrene).

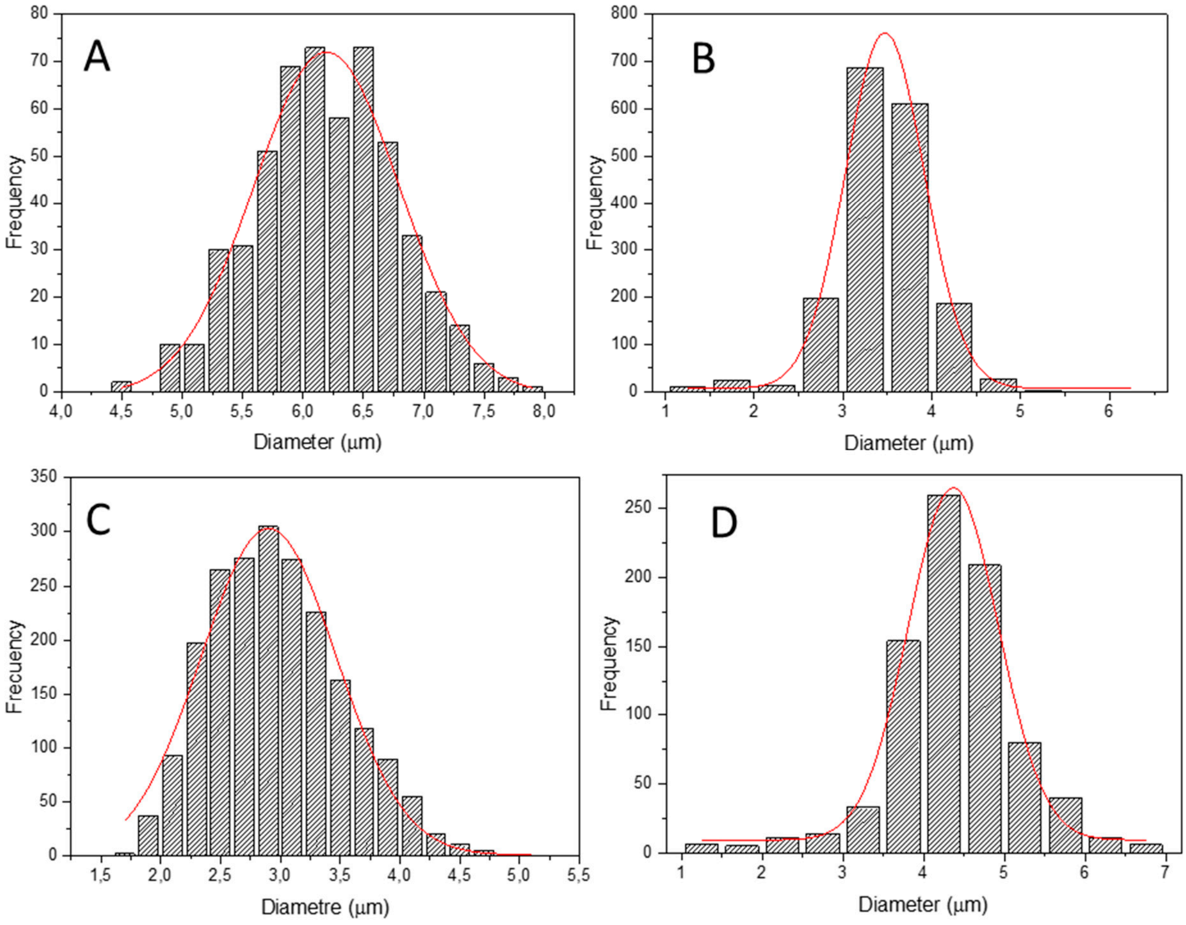


Figure S2. Pore size distribution in THF at 90%RH for: A) polystyrene, B) poly(2-chlorostryrene-co-styrene), C) poly(3-chlorostyrene-co-styrene) and D) poly(4-chlorostyrene-co-styrene).

Table S1. Glass transition temperatures of PS and P(xClS)s

| **Sample** | **Glass transition temperature (°C)** |
|---|---|
| Polystyrene | 105 |
| Poly(2-chlrostyrene) | 109 |
| Poly(3-chlorostyrene) | 84 |
| Poly(4-chlorostyrene) | 126 |

Table S2. Glass transition temperatures of P(S-co-xClS)s

| **Sample** | **Glass transition temperature (°C)** |
|---|---|
| Polystyrene | 105 |
| Poly(2-chlrostyrene-co-styrene) | 110 |
| Poly(3-chlorostyrene-co-styrene) | 99 |
| Poly(4-chlorostyrene-co-styrene) | 110 |

Table S3. Glass transition temperatures of PS/P(xClS) blends 90/10.

| Sample | Glass transition temperature (°C) |
|---|---|
| Polystyrene | 105 |
| PS/P2ClS | 89 |
| PS/P3ClS | 101 |
| PS/P4ClS | 90 |

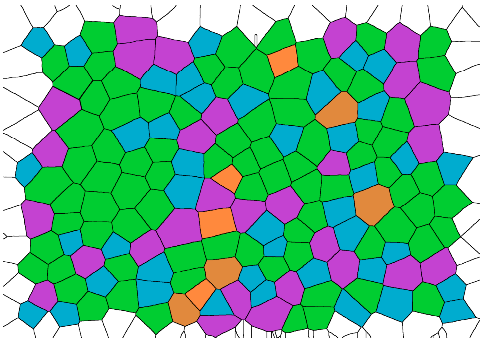

Figure S3. Voronoi tessellation for polystyrene/poly(3-chlorostyrene blend.(Orange=$P_4$, Blue=$P_5$, Green=$P_6$, Purple=$P_7$, Brown=$P_8$)
